# Regression-based normative data for Corsi Span and Supraspan learning and recall among Italian adults

**DOI:** 10.1007/s10072-024-07756-6

**Published:** 2024-09-09

**Authors:** Alessio Facchin, Sara Pegoraro, Mattia Rigoli, Ezia Rizzi, Veronica Strina, Sara Barera, Giulia Castiglieri, Roberta Daini, Chiara Guarnerio

**Affiliations:** 1 Deparment of Medical and Surgical Sciences, Magna Graecia University, Via Europa, Catanzaro, 88100 Italy; 2 Department of Psychology, University of Milano-Bicocca, Milan, 20126 Italy; 3 Milan Centre for Neuroscience (NeuroMI), Milano, Italy; 4 Neurological unit, ASST Valle Olona, Gallarate Hospital, Gallarate, Italy; 5 Università Cattolica del Sacro Cuore, Milan, 20123 Italy; 6 IRCCS Fondazione Don Carlo Gnocchi ONLUS, Milan, Italy

**Keywords:** Neuropsychological tests, Cognitive testing, Memory, Supraspan, Normative data, MLT

## Abstract

**Introduction:**

The Corsi Block Tapping Test, or Corsi Span (CS), is a widely used task to measure visuospatial short-term and working memory. The same setup can be used to administer the Corsi SupraSpan Learning (CSSL) and Recall (CSSR), tests assessing visuospatial long-term memory. While the CS has relatively recent normative data, those of the CSSL are outdated For CSSR, no normative data are available. Given this critical lack, our study aimed to provide updated norms for CS, CSSL, and specifically for the recall delayed phase (CSSR).

**Materials and methods:**

A sample of 340 healthy participants, aged between 20 and 89, took part in the study. Norms were developed using a regression approach and defined using rank equivalent scores and percentiles.

**Results:**

Age and education influenced Corsi’s Span, while SupraSpan Learning and Recall were influenced by age, education, and span. The comparison with previous norms for Span and SupraSpan Learning shows a high level of agreement.

**Conclusions:**

This study provides integrated norms to evaluate visuospatial memory in all aspects of immediate recall, long-term learning and delayed recall. Its use is needed to assess specific neuropsychological deficits, dissociate visuospatial versus verbal memory deficits and allow the evaluation of memory in patients with limited verbal abilities.

## Introduction

Developed by Philip Corsi in 1973, the Corsi Block Tapping Task has been used as a measure of visuospatial memory span in clinical and experimental settings for decades [1, 2]. In neuropsychological research, indeed, it is the most relevant and used non-verbal task and, in clinical assessment, it has proved to be a valid and reliable instrument to measure an individual’s visuospatial short-term and working memory [3–5].

In its classical version, the task consists of nine identical blocks on a board, and participants are asked to reproduce block-tapping sequences of increasing length. The repetition of the examiner’s sequence could be in the same (forward condition) or reverse order (backward condition). The longest sequence correctly reproduced in the former condition is the span, a measure of short-term memory storage capacity, whereas the one in the latter is a measure of visuospatial working memory. Aside from the table task, the test is also available in modified forms included in neuropsychological batteries [6], in a computerised [7] and touchscreen [8] format, and as a “walking Corsi” [9].

The Corsi span (CS) in Italy has relatively recent norms [10] and is frequently used to assess patients with different neurological conditions: Alzheimer’s disease [11], Parkinson’s disease [12], psychotic disorders [8] and traumatic brain injury [13].

The majority of studies have focused on the backward CS, as a reliable measure of visuospatial working memory and one of the measures of executive functions [2, 14], although in the clinical setting both forward and backward conditions are administered to almost all neurological patients.

The same setup of CS can be used to administer the Corsi SupraSpan Learning (CSSL) and Recall (CSSR), a test assessing visuospatial long-term learning and visuospatial long-term memory skills. This test, although less used in clinical practice, as well as in research, has undoubted advantages. First of all, it can be used to assess learning and long-term memory skills in patients with limited verbal abilities. Moreover it can be used to identify a dissociation between visuospatial and verbal memory deficits. Last but not least, it has a great sensitivity even to subtle long-term memory deficits [15–17], having the chance to be linked to the individual span.

Various supraspan techniques have been developed to assess nonverbal material acquisition. The CSSL procedure, scoring system, and normative values were first introduced in Italy by Spinnler and Tognoni [18]. Subjects learned a fixed sequence of eight block touches, which were repeated until they achieved a specific criterion (three consecutive sequences repeated correctly) or 18 presentations.

The previously published procedure, however, had one limitation, as pointed out by Capitani et al. [19]: an individual with a low short-term memory span may find it more difficult to learn a fixed-length sequence, while a long spatial sequence may be affected by a short-term memory span (CS). Consequently, Capitani and colleagues [19] provide separate CSSL norms that take into account the CS length for values ranging from 4 to 6.

According to the authors, CSSL administration is not possible if the CS score is lower than three or equal to eight, since the sequence length itself is equal to eight cubes. In other words, CSSL cannot be administered when the CS is too small or too big. However, this criterion does not consider that the memory systems involved in CS and CSSL are different and double-dissociated [20, 21]. As a consequence, a specific visual-spatial short-term memory impairment may impact CS but not CSSL. Moreover, in the case of a CS score of eight, the procedure for CSSL requires immediate repetition of the sequence almost three times to reach the highest score. Consequently, whoever gets a CS of 8 could fail CSSL.

This administration limit, reported by Capitani and colleagues [19], can be resolved by adjusting CSSL scores based on CS. On the other hand CSSL score could be adjusted for CS score, in addition to the other classical demographic variables (age, education and sex), using a single regression model instead of having a different set of norms.

An additional uncertain point in both previous CSSL studies is related to the recall of the learned sequence, named Corsi SupraSpan Recall (CSSR). The test procedure proposed by the authors included a recall part, but without reporting normative data in both publications [18, 19]. Having normative data available for recall would mean making the task able to provide a measure of long-term visuospatial memory, useful, for example, in patients with limited verbal abilities.

Therefore, the aim of this study was to update CSSL norms and, more importantly, to provide normative data for CSSR. Since CSSL seems to be influenced by CS, normative data for CS, based on the same sample, were also provided, as well as the relationship between the two tasks. Finally, CS and CSSL normative data will be compared to the available norms.

## Materials and methods

### Participants

A power analysis was performed to assess the minimum sample size required. Based on a regression model with four independent demographic variables (age, education, sex and CS), using α = 0.05, power = 0.80 and a small effect size f^2^ = 0.04, a minimum sample of 304 participants was required. Participants were native Italians without any history of neurological or psychiatric disorders, current or past (including stroke, brain injury, clinically diagnosed dementia, depression, alcohol or drug abuse), and obtained a normal score on the Mini-Mental State Examination-MMSE (adjusted score > 23.8) [22]. Each participant had normal or corrected-to-normal vision. A convenience sample of volunteers was selected among those who could be directly contacted by the different examiners. No compensation was provided. Initially, a sample of 342 participants was collected, but two participants did not meet the inclusion criteria (lower MMSE score) and consequently, a final sample of 340 participants was obtained (177 female, 163 male; mean age = 51.6, SD = 19.4, range 21–89; Education mean = 13.1, SD = 4.6, range 4–25). The subdivision of participants by age, education and sex is visible in the supplementary materials table A1 at https://osf.io/jcn9d/. Informed consent was signed by participants before the evaluation. This study was performed in line with the principles of the Declaration of Helsinki. Approval was granted by the Ethics Committee of Milano Bicocca University (RM-2022-493; 7 February 2022).

### Corsi block tapping board

**Fig. 1:**
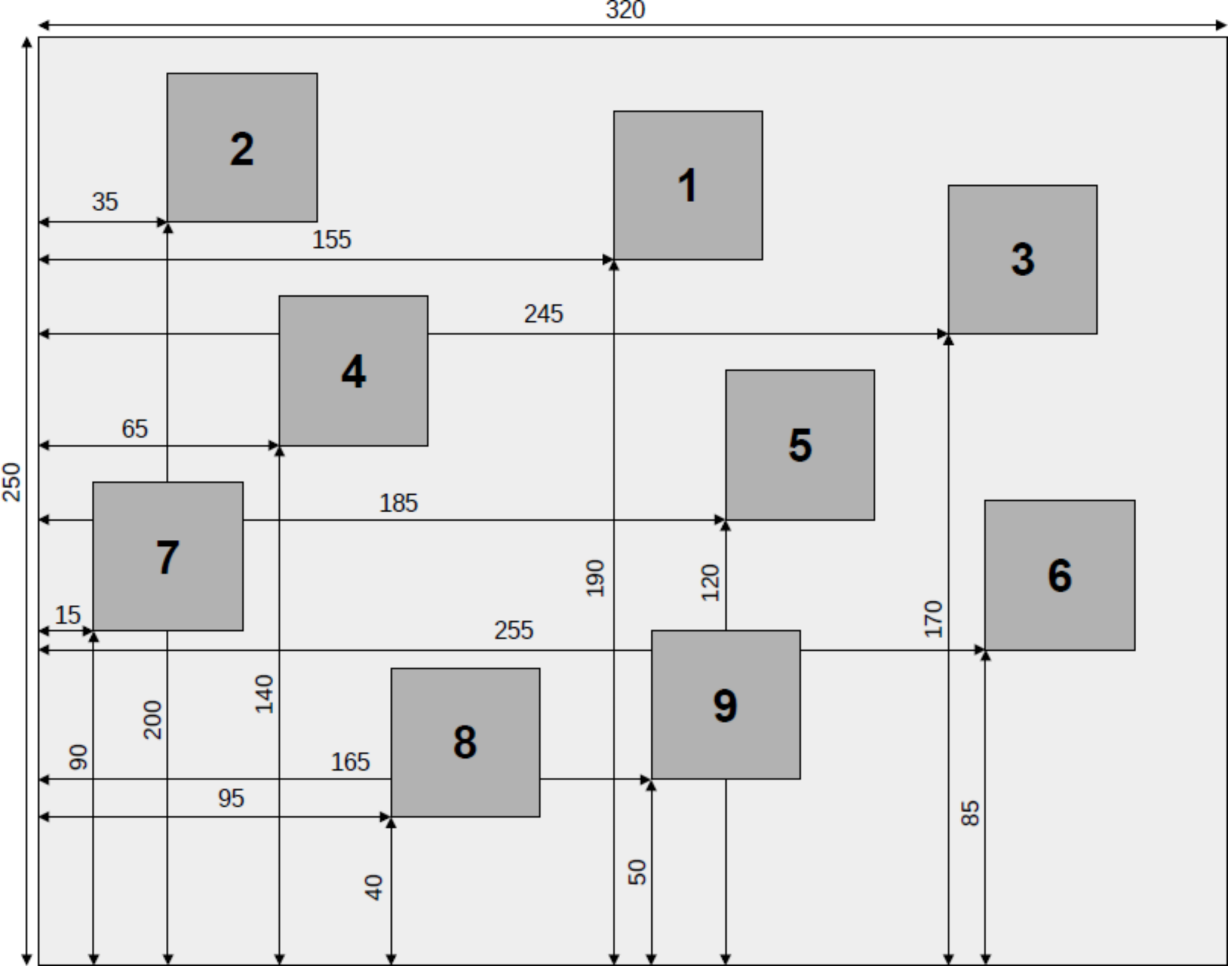
Physical measures of the apparatus used for Corsi Span block tapping test and sorsi supraSpan learning and recall test

The apparatus for the task consisted of a set of nine cubes arranged irregularly. Since different versions of the apparatus were described [1, 23] and the performance depends on path configuration [24], the apparatus is described in detail. The block tapping table consists of a white wooden board of 320 × 250 mm with nine white cubes with 40 mm edges positioned in a pseudo random order as visible in Fig. 1. The digits 1 through 9 were placed on the examiner side of the cubes and numbered as reported in the figure. Examiner side was at the bottom part of the figure and the participant’s side at the top side. The same board was used for all tests.

### Tests and procedure

#### Corsi span

The procedure is derived from the one presented by Orsini et al. [25]. The examiner sits at a desk in front of the participant and says: “*Touch the cubes that I touch*,* in the same order*”. With the forefinger of the dominant hand the examiner touches in sequence the cubes indicated on the notation protocol (Table A2 at https://osf.io/jcn9d/), one cube every two seconds returning to the space between himself/herself and the board after each cube touched.

For each block there are three sequences of cubes. Each block, compared to the previous one, consists of an increasing number of cubes to touch. In order to pass the block sequence, the subject must correctly execute two out of three trials. The span is given by the block in which the subject provided at least two correct answers. Since the performance at CS depends on path configuration [24], the paths used are standard and derived from the first three series from Orsini et al. [25] with a modification on the sequence with length of 8: the sequence identical to CSSL was not used.

#### Corsi supraSpan learning and recall

The examiner presents the fixed series of eight touches (5-8-3-2-6-7-1-9) at the rate of one cube every two seconds. The examiner should keep his or her hand between the table and the examiner after each touch. The examiner says: “*Now we will do the same thing as before*,* but I will touch a greater number of cubes. You will observe carefully and when I have finished*,* I ask you to touch the same cubes that I touched*,* in the same order*”. The examiner notes the sequence of cubes touched for each attempt, considering only the first eight touches. If the participant reproduces the sequence correctly, he/she is asked to reproduce it again without repetition from the examiner. The test ends when the criterion of three consecutive correct repetitions is reached, or after 18 attempts. The examiner does not say that the sequence is identical trial by trial. The examiner does not say that another attempt will be required later. After five minutes, with no interfering visuo-spatial task, a recall of the previously presented sequence is asked (CSSR). The examiner says: “*Now I ask you to reproduce the sequence of cubes that we have reproduced several times before*”. The examiner notes the sequence of cubes touched, considering only the first eight. The scoring system was based on that published by Capitani et al. [19]. Each response was scored based on the probability that the correct response would be given by chance (or at least a partial chance). Table A3 at https://osf.io/jcn9d/ shows the corresponding partial scores for each response. If participants achieved the criterion prior to the 18th trial, they received a score corresponding to the correct performance (1.62) for the remaining trials up to the 18th. Consequently, CSSL had a maximum score of 29.16 and CSSR of 1.62.

### Statistical analyses

The analyses were then divided into three sections. As a first step, descriptive analyses were conducted along with correlations between demographic variables and raw scores. Normative values were defined in the second part. The regression-based procedure definition of norms was used, which includes several consecutive steps in line with different neuropsychological tests [26–28] and described in detail elsewhere [29]. To find the most appropriate transformation of demographic variables (age, education and sex) on the dependent variables (CS, CSSL and CSSR, taken separately) the general linear model was used. A series of bivariate regressions were compared based on the lowest Akaike’s Information Criterion (AIC) [30]. The most effective transformations of demographic independent variables were selected. They were included in a series of bivariate and multivariate regressions with one to four predictors. Based on the small AIC, the most appropriate regression model was then selected, if it was significant (*p* < 0.05). The same best regression model was then applied to deviations from the mean scores for independent variables (in their appropriate transformations) and dependent variables. Reversing regression coefficients of these last regressions, a correction regression equation was obtained. Based on the correction regression, a score grid correction was developed. Adjusted scores for demographic variables were obtained by adding the correction score to the raw scores. On these adjusted times, the one-sided nonparametric 95% tolerance limits, with a confidence interval of 95% were calculated. Percentile ranks and rank-based equivalent scores on the adjusted score were calculated [28, 31] (Table 1).

**Table 1:** Descriptive statistics (mean, SD and range) for demographic variables and the three scores of the tests (CS, CSSL and CSSR) together with their correlations. * *p* < 0.05; ** *p* < 0.01; *** *p* < 0.001

	Mean (SD)	Range	Age	Education	sex	CS	CSSL
Age	51.6 (19.4)	21–89					
Education	13.1 (4.6)	4–25	-0.6***				
sex	177 F/163 M		-0.07	0.07			
CS	4.97 (1.03)	2–8	-0.46***	0.45***	0.03		
CSSL	18.67 (7.18)	1.56–29.16	-0.45***	0.49***	0.08	0.47***	
CSSR	1.14 (0.54)	0.06–1.62	-0.40***	0.38***	0.04	0.36***	0.61***

In the third part, in order to compare the diagnostic performance of the normative data here defined to the more recent norms available for CS and CSSL [10, 19], a simulation analysis was performed based on a generated dataset of memory impaired patients. This population was chosen for the comparison because data for CS and CSSL are both needed. The same procedure was used in a previous comparison of norms via simulation [32]. By this way, to compare norms, it is sufficient to use published means and standard deviations, overcoming some problems that were encountered when comparing different normative scoring systems only descriptively. Different normative diagnostic systems rely on different adjustment regression equations that were derived from data collected in different times (2013 vs. 2023 for CS and 1990 vs. 2023 for CSSL) and cannot be compared directly. In addition, in order to compare the classification abilities of different normative systems, one must utilise a sample that is independent from the sample used for the development of the normative system. This permits to generalise predictions to unseen data and most importantly, the characteristics of the sample used for comparison reflect at better the characteristics of a clinical sample. Specifically, classification accuracy and area under the curve (AUC) were used to compare diagnostic performance between normative scoring systems. The agreement was assessed using Cohen’s Kappa. All analyses were performed using the R statistical environment 4.3.2 and specific packages [33]. Supplementary materials are available at https://osf.io/jcn9d/.

## Results

### Descriptive statistics

The descriptive mean results and correlation on the three scores (CS, CSSL and CSSR) among the three demographic indicators are reported in Table 1. Mean results and SD on CS, CSSL and CSSR separated for decades of ages are reported in supplementary table A4, available at https://osf.io/jcn9d/. Results showed a negative moderate correlation between Age and CS, CSSL and CSSR; a moderate correlation between Education and CS and CSSL, but more importantly, a moderate correlation between CS and CSSL, a moderate to low correlation between CS and CSSR and a moderate to high correlation between CSSL and CSSR.

Considering the lower (2 and 3) and higher score (8) on CS, in the sample collected, the CSSL and CSSR scores were then described. Two participants out of 340 obtained a raw score of 2 on CS (0.6%), and five out of 340 participants obtained a score of 3 (0.9%). The score of 8 was obtained by 3 participants out of 340 (0.9%) and no one of these participants reached the maximum score of 29.16 in CSSL. Conversely, CSSL maximum score was reached by two participants (0.6%). Consequently, the *ceiling* and *floor* effects are marginal in this group of healthy participants. Afterall, using the normative data defined, the CSSL score of participants who showed a pathological score on CS, no one exhibited a pathological score in CSSL and CSSR. Again, we tested the reversed hypothesis, observing the CS score on CSSL pathological score participants. No one exhibited a pathological score in CS. Finally, 161 participants (47.4%) reached a ceiling effect on CSSR and 23 (6.8%) a floor effect. These results indicate that the performance on CS and CSSL are double dissociated, almost in the healthy controls participants tested.

### Normative data definition

The bivariate and multivariate regressions selection using the AIC method showed that the model that better describes CS score include Age in its logarithmic transformation and Education in its square root transformation. CSSL was influenced by Age and Education both in their square root transformation and most importantly by CS using the square root transformation. CSSR was also influenced by Age and Education in square root and inverse transformation respectively and importantly by CS. AIC tables used for selecting the best models are shown in Table A5 available at https://osf.io/jcn9d/.

In order to obtain correction values, multiple regressions on the raw scores were redrawn from deviations from mean scores and their coefficients reversed. The regression for obtaining adjusted scores are reported in the caption of Tables 2, 3 and 4 for CS, CSSL and CSSR respectively. The *R*^*2*^ for each regression were 0.24, 0.34 and 0.22 respectively.

The correction grids derived from these regression equations are available in Tables 2, 3 and 4 for quick and easy application in clinical settings. The adjusted score can be calculated by adding the raw score to the reported value obtained by the table or by regressions.

**Table 2:** Correction grid for computing the adjusted scores of Corsi Span (CS). The adjusted score can be calculated by adding the raw score to the reported value obtained by the table. Age and education should be selected based on the nearest values. If precise scoring is required, the correction regression should be used. Adjusted score = raw score + 0.7616*(log(Age)-3.864) − 0.4229*(sqrt(Education)-3.555). The logarithms were intended to be computed on the natural base *e*

Edu/Age	20	25	30	35	40	45	50	55	60	65	70	75	80	85
5	-0.10	0.06	0.21	0.32	0.42	0.51	0.59	0.67	0.73	0.79	0.85	0.90	0.95	1.00
8	-0.35	-0.18	-0.05	0.07	0.17	0.26	0.34	0.42	0.48	0.54	0.6	0.65	0.7	0.75
13	-0.68	-0.51	-0.37	-0.26	-0.15	-0.07	0.02	0.09	0.15	0.22	0.27	0.32	0.37	0.42
16		-0.68	-0.54	-0.42	-0.32	-0.23	-0.15	-0.08	-0.01	0.05	0.1	0.16	0.21	0.25
18		-0.78	-0.64	-0.53	-0.42	-0.33	-0.25	-0.18	-0.12	-0.05	0	0.05	0.1	0.15
21			-0.79	-0.67	-0.57	-0.48	-0.4	-0.33	-0.26	-0.2	-0.14	-0.09	-0.04	0.01

**Table 3:** Correction grid for computing the adjusted scores of CSSL based on Age, Education and Corsi Span size. The adjusted score can be calculated by adding the raw score to the reported value obtained by the table. Age and education should be selected based on the nearest values

Span	Edu/Age	20	25	30	35	40	45	50	55	60	65	70	75	80	85
	5	8.72	9.16	9.56	9.93	10.27	10.6	10.9	11.19	11.47	11.74	11.99	12.24	12.48	12.71
2	8	7.03	7.47	7.87	8.24	8.58	8.91	9.21	9.5	9.78	10.05	10.3	10.55	10.79	11.02
	13	4.81	5.25	5.66	6.02	6.37	6.69	7.00	7.29	7.56	7.83	8.09	8.33	8.57	8.80
	16		4.13	4.53	4.90	5.24	5.57	5.87	6.16	6.44	6.70	6.96	7.21	7.45	7.68
	18		3.44	3.84	4.21	4.55	4.87	5.18	5.47	5.75	6.01	6.27	6.52	6.75	6.99
	21			2.87	3.24	3.58	3.90	4.21	4.50	4.78	5.04	5.30	5.55	5.79	6.02
Span	Edu/Age	20	25	30	35	40	45	50	55	60	65	70	75	80	85
3	5	5.90	6.35	6.75	7.12	7.46	7.78	8.09	8.38	8.66	8.92	9.18	9.42	9.66	9.89
	8	4.21	4.66	5.06	5.43	5.77	6.09	6.40	6.69	6.97	7.23	7.49	7.73	7.97	8.20
	13	2.00	2.44	2.84	3.21	3.55	3.88	4.18	4.47	4.75	5.02	5.27	5.52	5.76	5.99
	16		1.31	1.72	2.09	2.43	2.75	3.06	3.35	3.62	3.89	4.15	4.39	4.63	4.86
	18		0.62	1.02	1.39	1.74	2.06	2.36	2.65	2.93	3.20	3.45	3.70	3.94	4.17
	21			0.05	0.42	0.77	1.09	1.40	1.69	1.96	2.23	2.48	2.73	2.97	3.20
Span	Edu/Age	20	25	30	35	40	45	50	55	60	65	70	75	80	85
4	5	3.53	3.97	4.37	4.74	5.09	5.41	5.71	6.01	6.28	6.55	6.80	7.05	7.29	7.52
	8	1.84	2.28	2.68	3.05	3.40	3.72	4.03	4.32	4.59	4.86	5.11	5.36	5.6	5.83
	13	-0.38	0.07	0.47	0.84	1.18	1.50	1.81	2.10	2.38	2.64	2.90	3.15	3.38	3.62
	16		-1.06	-0.66	-0.29	0.06	0.38	0.68	0.97	1.25	1.52	1.77	2.02	2.26	2.49
	18		-1.75	-1.35	-0.98	-0.64	-0.31	-0.01	0.28	0.56	0.83	1.08	1.33	1.57	1.80
	21			-2.32	-1.95	-1.61	-1.28	-0.98	-0.69	-0.41	-0.14	0.11	0.36	0.60	0.83
Span	Edu/Age	20	25	30	35	40	45	50	55	60	65	70	75	80	85
5	5	1.44	1.88	2.28	2.65	3.00	3.32	3.62	3.91	4.19	4.46	4.71	4.96	5.20	5.43
	8	-0.25	0.19	0.59	0.96	1.31	1.63	1.93	2.23	2.50	2.77	3.02	3.27	3.51	3.74
	13	-2.47	-2.02	-1.62	-1.25	-0.91	-0.59	-0.28	0.01	0.29	0.55	0.81	1.06	1.29	1.53
	16		-3.15	-2.75	-2.38	-2.03	-1.71	-1.41	-1.12	-0.84	-0.57	-0.32	-0.07	0.17	0.40
	18		-3.84	-3.44	-3.07	-2.73	-2.40	-2.10	-1.81	-1.53	-1.26	-1.01	-0.76	-0.52	-0.29
	21			-4.41	-4.04	-3.7	-3.37	-3.07	-2.78	-2.50	-2.23	-1.98	-1.73	-1.49	-1.26
Span	Edu/Age	20	25	30	35	40	45	50	55	60	65	70	75	80	85
6	5	-0.45	-0.01	0.39	0.76	1.11	1.43	1.73	2.02	2.30	2.57	2.82	3.07	3.31	3.54
	8	-2.14	-1.70	-1.30	-0.93	-0.58	-0.26	0.05	0.34	0.61	0.88	1.13	1.38	1.62	1.85
	13	-4.36	-3.91	-3.51	-3.14	-2.80	-2.48	-2.17	-1.88	-1.60	-1.34	-1.08	-0.83	-0.60	-0.36
	16		-5.04	-4.64	-4.27	-3.92	-3.60	-3.30	-3.01	-2.73	-2.46	-2.21	-1.96	-1.72	-1.49
	18		-5.73	-5.33	-4.96	-4.62	-4.29	-3.99	-3.70	-3.42	-3.15	-2.90	-2.65	-2.41	-2.18
	21			-6.30	-5.93	-5.59	-5.26	-4.96	-4.67	-4.39	-4.12	-3.87	-3.62	-3.38	-3.15
Span	Edu/Age	20	25	30	35	40	45	50	55	60	65	70	75	80	85
7	5	-2.19	-1.75	-1.34	-0.97	-0.62	-0.31	0.00	0.29	0.56	0.83	1.09	1.33	1.57	1.80
	8	-3.88	-3.43	-3.03	-2.66	-2.32	-2.00	-1.69	-1.40	-1.13	-0.86	-0.60	-0.36	-0.12	0.11
	13	-6.09	-5.65	-5.25	-4.88	-4.54	-4.21	-3.91	-3.62	-3.34	-3.08	-2.82	-2.57	-2.33	-2.10
	16		-6.78	-6.37	-6.01	-5.66	-5.34	-5.03	-4.74	-4.47	-4.20	-3.94	-3.70	-3.46	-3.23
	18		-7.47	-7.07	-6.70	-6.35	-6.03	-5.73	-5.44	-5.16	-4.89	-4.64	-4.39	-4.15	-3.92
	21			-8.04	-7.67	-7.32	-7.00	-6.70	-6.41	-6.13	-5.86	-5.61	-5.36	-5.12	-4.89
Span	Edu/Age	20	25	30	35	40	45	50	55	60	65	70	75	80	85
8	5	-3.81	-3.36	-2.96	-2.59	-2.25	-1.93	-1.62	-1.33	-1.05	-0.79	-0.53	-0.28	-0.05	0.19
	8	-5.50	-5.05	-4.65	-4.28	-3.94	-3.62	-3.31	-3.02	-2.74	-2.48	-2.22	-1.97	-1.74	-1.50
	13	-7.71	-7.27	-6.87	-6.50	-6.15	-5.83	-5.53	-5.24	-4.96	-4.69	-4.44	-4.19	-3.95	-3.72
	16		-8.39	-7.99	-7.62	-7.28	-6.96	-6.65	-6.36	-6.08	-5.82	-5.56	-5.32	-5.08	-4.84
	18		-9.09	-8.68	-8.32	-7.97	-7.65	-7.34	-7.05	-6.78	-6.51	-6.25	-6.01	-5.77	-5.54
	21			-9.65	-9.28	-8.94	-8.62	-8.31	-8.02	-7.75	-7.48	-7.22	-6.98	-6.74	-6.51

If precise scoring is required, the correction regression reported should be used. Adjusted score = raw score + 0.841 * (sqrt(age) − 7.044) − 2.852 * (sqrt(education) − 3.555) − 8.855 * (sqrt(span) − 2.218)

**Table 4:** Correction grid for computing the adjusted scores of CSSR based on Age, Education and Corsi Span size. The adjusted score can be calculated by adding the raw score to the reported value obtained by the table. Age and education should be selected based on the nearest values

Span	Edu/Age	20	25	30	35	40	45	50	55	60	65	70	75	80	85
2	5	0.48	0.52	0.56	0.59	0.62	0.65	0.68	0.70	0.73	0.75	0.78	0.80	0.82	0.84
	8	0.36	0.40	0.43	0.47	0.50	0.52	0.55	0.58	0.60	0.63	0.65	0.67	0.69	0.71
	13	0.23	0.27	0.30	0.33	0.36	0.39	0.42	0.45	0.47	0.49	0.52	0.54	0.56	0.58
	16		0.21	0.24	0.28	0.31	0.34	0.36	0.39	0.41	0.44	0.46	0.48	0.50	0.52
	18		0.18	0.21	0.25	0.28	0.30	0.33	0.36	0.38	0.41	0.43	0.45	0.47	0.49
	21			0.17	0.20	0.23	0.26	0.29	0.32	0.34	0.36	0.39	0.41	0.43	0.45
Span	Edu/Age	20	25	30	35	40	45	50	55	60	65	70	75	80	85
3	5	0.29	0.33	0.36	0.39	0.42	0.45	0.48	0.51	0.53	0.55	0.58	0.60	0.62	0.64
	8	0.16	0.20	0.23	0.27	0.30	0.33	0.35	0.38	0.40	0.43	0.45	0.47	0.49	0.51
	13	0.03	0.07	0.10	0.13	0.17	0.19	0.22	0.25	0.27	0.29	0.32	0.34	0.36	0.38
	16		0.01	0.05	0.08	0.11	0.14	0.16	0.19	0.22	0.24	0.26	0.28	0.30	0.33
	18		-0.02	0.01	0.05	0.08	0.11	0.13	0.16	0.18	0.21	0.23	0.25	0.27	0.29
	21			-0.03	0.00	0.04	0.06	0.09	0.12	0.14	0.17	0.19	0.21	0.23	0.25
Span	Edu/Age	20	25	30	35	40	45	50	55	60	65	70	75	80	85
4	5	0.14	0.18	0.22	0.25	0.28	0.31	0.34	0.36	0.39	0.41	0.44	0.46	0.48	0.50
	8	0.02	0.06	0.09	0.12	0.16	0.18	0.21	0.24	0.26	0.29	0.31	0.33	0.35	0.37
	13	-0.11	-0.08	-0.04	-0.01	0.02	0.05	0.08	0.11	0.13	0.15	0.18	0.20	0.22	0.24
	16		-0.13	-0.10	-0.06	-0.03	0.00	0.02	0.05	0.07	0.10	0.12	0.14	0.16	0.18
	18		-0.16	-0.13	-0.09	-0.06	-0.04	-0.01	0.02	0.04	0.07	0.09	0.11	0.13	0.15
	21			-0.17	-0.14	-0.11	-0.08	-0.05	-0.02	0.00	0.02	0.05	0.07	0.09	0.11
Span	Edu/Age	20	25	30	35	40	45	50	55	60	65	70	75	80	85
5	5	0.03	0.07	0.11	0.14	0.17	0.20	0.23	0.25	0.28	0.30	0.33	0.35	0.37	0.39
	8	-0.09	-0.05	-0.02	0.02	0.05	0.07	0.10	0.13	0.15	0.18	0.20	0.22	0.24	0.26
	13	-0.22	-0.18	-0.15	-0.12	-0.09	-0.06	-0.03	0.00	0.02	0.04	0.07	0.09	0.11	0.13
	16		-0.24	-0.21	-0.17	-0.14	-0.11	-0.09	-0.06	-0.04	-0.01	0.01	0.03	0.05	0.07
	18		-0.27	-0.24	-0.20	-0.17	-0.15	-0.12	-0.09	-0.07	-0.04	-0.02	0.00	0.02	0.04
	21			-0.28	-0.25	-0.22	-0.19	-0.16	-0.13	-0.11	-0.09	-0.06	-0.04	-0.02	0.00
Span	Edu/Age	20	25	30	35	40	45	50	55	60	65	70	75	80	85
6	5	-0.05	-0.02	0.02	0.05	0.08	0.11	0.14	0.17	0.19	0.21	0.24	0.26	0.28	0.30
	8	-0.18	-0.14	-0.11	-0.07	-0.04	-0.01	0.01	0.04	0.06	0.09	0.11	0.13	0.15	0.17
	13	-0.31	-0.27	-0.24	-0.21	-0.18	-0.15	-0.12	-0.09	-0.07	-0.05	-0.02	0.00	0.02	0.04
	16		-0.33	-0.29	-0.26	-0.23	-0.20	-0.18	-0.15	-0.13	-0.10	-0.08	-0.06	-0.04	-0.02
	18		-0.36	-0.33	-0.29	-0.26	-0.23	-0.21	-0.18	-0.16	-0.13	-0.11	-0.09	-0.07	-0.05
	21			-0.37	-0.34	-0.31	-0.28	-0.25	-0.22	-0.20	-0.18	-0.15	-0.13	-0.11	-0.09
Span	Edu/Age	20	25	30	35	40	45	50	55	60	65	70	75	80	85
7	5	-0.13	-0.09	-0.06	-0.02	0.01	0.04	0.06	0.09	0.11	0.14	0.16	0.18	0.20	0.22
	8	-0.26	-0.22	-0.18	-0.15	-0.12	-0.09	-0.06	-0.04	-0.01	0.01	0.03	0.06	0.08	0.10
	13	-0.39	-0.35	-0.31	-0.28	-0.25	-0.22	-0.20	-0.17	-0.14	-0.12	-0.10	-0.08	-0.06	-0.03
	16		-0.41	-0.37	-0.34	-0.31	-0.28	-0.25	-0.23	-0.20	-0.18	-0.15	-0.13	-0.11	-0.09
	18		-0.44	-0.40	-0.37	-0.34	-0.31	-0.28	-0.26	-0.23	-0.21	-0.19	-0.16	-0.14	-0.12
	21			-0.44	-0.41	-0.38	-0.35	-0.33	-0.30	-0.27	-0.25	-0.23	-0.21	-0.19	-0.16
Span	Edu/Age	20	25	30	35	40	45	50	55	60	65	70	75	80	85
8	5	-0.20	-0.12	-0.12	-0.09	-0.06	-0.03	0.00	0.02	0.05	0.07	0.10	0.12	0.14	0.16
	8	-0.32	-0.28	-0.25	-0.22	-0.18	-0.16	-0.13	-0.1	-0.08	-0.06	-0.03	-0.01	0.01	0.03
	13	-0.45	-0.42	-0.38	-0.35	-0.32	-0.29	-0.26	-0.23	-0.21	-0.19	-0.16	-0.14	-0.12	-0.10
	16		-0.47	-0.44	-0.4	-0.37	-0.34	-0.32	-0.29	-0.27	-0.24	-0.22	-0.20	-0.18	-0.16
	18		-0.50	-0.47	-0.44	-0.40	-0.38	-0.35	-0.32	-0.30	-0.27	-0.25	-0.23	-0.21	-0.19
	21			-0.51	-0.48	-0.45	-0.42	-0.39	-0.36	-0.34	-0.32	-0.29	-0.27	-0.25	-0.23

If precise scoring is required, the correction regression should be used. Adjusted score = raw score + 0.0747 * (sqrt(age) − 7.044) − 0.271*(log(education) − 2.497) − 0.491 * (log(span) − 1.582). The logarithms were intended to be computed on the natural base *e*

 All the adjusted scores were not normally distributed (all *ps* < 0.01), consequently, the one-side inner (ITL) and outer (OTL) 95% tolerance limits with 95% confidence intervals were calculated using a non-parametrical approach. For a sample of 340 participants, using a score in which the higher, the better, they correspond to the 11th and 24th observations. Their values are reported in table 5

**Table 5:** Equivalent scores together with outer (OTL) and inner (ITL) tolerance limits for the three tests based on the adjusted score. CS = Corsi Span; CSSL, Corsi SupraSpan Learning; CSSR Corsi SupraSpan Recall

	Equivalent score						
Test	0	1	2	3	4	OTL	ITL
CS	≤ 3.43	3.44–4.21	4.22–4.52	4.53–4.97	> 4.97	3.43	3.7
CSSL	≤ 6.75	6.76–12.67	12.68–16.63	16.64–19.37	> 19.37	6.75	9.93
CSSR	≤ 0.16	0.17–0.65	0.66–0.97	0.98–1.24	> 1.24	0.16	0.37

The cut-off scores provided by the outer tolerance limits, together with median and other intermediate intervals were subsequently transformed into rank-based Equivalent Scores (ES). Moreover, for each score, percentiles were calculated. They are listed in Table 5 and A6 available at https://osf.io/jcn9d/, respectively. To facilitate the scoring process, an Excel spreadsheet was available at the aforementioned link.

### Comparison between norms

In order to compare the defined norms to those already available in the literature, a simulated sample of 100 memory impaired participants was created. The means and standard deviations of the simulated sample were retrieved from Cosi et al. [34] considering 50 participants with mild memory impairment and 50 participants with memory impairment as defined in the study. Since CS and CSSL were related, their values were assigned pseudo-randomly to allow them to correlate positively (*r* = 0.22, *p* < 0.01) as reported in the study. Since the previous CSSL norms does not take into account lower scores on CS (2 and 3), comparison of CSSL was performed only on a small number of simulated cases (43). Confusion matrices of simulated patients classification were reported in Table A7, performance metrics in Table A8 in the online supplementary materials, available with generated dataset and R script at https://osf.io/jcn9d/. Results of norms comparisons show a high agreement between old and new norms for CS and CSSL (accuracy about 0.90), confirming the similarity of both norms, even though for CSSL previous norms were developed more than 30 years ago also using different procedures.

## Discussion

The aim of this study was to define updated normative data for CS, CSSL together with CSSR, considering also the effect of CS on these latter. For this purpose, the performance of a group of 340 healthy adult individuals were analysed in relation to socio-demographic variables, such as age, sex and education; CSSL and CSSR were analysed in regard to CS score, too.

As expected from previous studies [10, 18, 19], in all tasks increasing age was associated with progressively lower performance, while higher education was related to a better performance. Sex did not significantly influence performance on any task, as found by Capitani and colleagues [19] and in line with other several studies showing the absence of sex differences in visuospatial memory [9, 35]. Piccardi and collaborators [36], indeed, suggested the importance of correcting CS scores for education more than for sex, since age and education regulate sex effects. More importantly, CSSL and CSSR are both influenced by CS. By adjusting CSSL and CSSR norms according to various CS levels, the issues highlighted by Capitani and colleagues [19] could be solved.

Comparing norms, the CS and CSSL norms exhibited strong agreement in terms of detecting the absence or the presence of a memory deficit, even though the CSSL norms were developed over 30 years ago. This result is not a given one. While previous research has demonstrated satisfactory agreement between old and recent norms across various verbal memory tests [33], the level of concordance observed in this investigation is notably high.

However, our norms differ from the previous norms in two aspects. Firstly, they incorporate CS’s role in CSSL using a single regression model. Secondly, the norms expand the range of CS between two and eight, bypassing the limitations of Capitani [19]. The updated normative data for CS and CSSL and the computation of those of CSSR, not previously available, represents a step forward for the neuropsychological practice; this latter aspect, in particular, should allow the reliable assessment of the performance in visuospatial learning and long-term visuospatial memory also for those neuropsychological patients with limited verbal abilities. Using these tasks would enable the assessment of how visuo-spatial memory deficits impact the functioning of other cognitive functions as well. Furthermore, this contribution clarifies the administration methods of these instruments, making their execution more uniform among clinicians and researchers.

Considering the various applications of these instruments, such as clinical diagnosis, treatment monitoring, and research, this contribution also clarifies their administration methods. This would make their execution more uniform in different fields.

A limitation of the study or in general to the paradigm of CS/CSSL/CSSR is the insufficient number of studies on validity and reliability. CS has been validated in WISC-III [37] and with a factor analysis [38], its split-half reliability was 0.75 [39], and the test-retest reliability was between 0.70 and 0.79 [37]. CSSL demonstrated its ecological validity in a study in which navigation abilities were predicted by CSSL as Alzheimer’s Disease progressed [40]. In our knowledge, only one piece is available for reliability of CSSL which shows an *r* = 0.80 [18]. The absence of data regarding the validity and reliability of these tests was reported in 1998 and remains valid to the present day [3]. All these psychometric measures should be evaluated in future studies.

The average level of education of many populations, including Italians, has increased in recent years. Therefore, updated normative data are required to reflect not only evolving cognitive abilities, but also demographic changes. The average educational level of the sample was found to differ from the previous norms: 13.1 years (current study) compared to 10.7 years [19]. Using outdated data could lead to incorrect interpretations of test results for individuals with higher education levels [41, 42], thus neuropsychological tests should be interpreted using current norms to allow for a more accurate assessment of cognitive functions and, in this particular case, of visuospatial working memory.
